# Palytoxin Found in *Palythoa* sp. Zoanthids (Anthozoa, Hexacorallia) Sold in the Home Aquarium Trade

**DOI:** 10.1371/journal.pone.0018235

**Published:** 2011-04-04

**Authors:** Jonathan R. Deeds, Sara M. Handy, Kevin D. White, James D. Reimer

**Affiliations:** 1 Center for Food Safety and Applied Nutrition, United States Food and Drug Administration, College Park, Maryland, United States of America; 2 Department of Chemistry, Biology, and Marine Science, University of the Ryukyus, Nishihara, Okinawa, Japan; Smithsonian's National Zoological Park, United States of America

## Abstract

Zoanthids (Anthozoa, Hexacorallia) are colonial anemones that contain one of the deadliest toxins ever discovered, palytoxin (LD_50_ in mice 300 ng/kg), but it is generally believed that highly toxic species are not sold in the home aquarium trade. We previously showed that an unintentionally introduced zoanthid in a home aquarium contained high concentrations of palytoxin and was likely responsible for a severe respiratory reaction when an individual attempted to eliminate the contaminant colonies using boiling water. To assess the availability and potential exposure of palytoxin to marine aquarium hobbyists, we analyzed zoanthid samples collected from local aquarium stores for palytoxin using liquid chromatography and high resolution mass spectrometry and attempted to identify the specimens through genetic analysis of 16S and cytochrome *c* oxidase 1 (COI) markers. We found four specimens of the same apparent species of zoanthid, that we described previously to be responsible for a severe respiratory reaction in a home aquarium, to be available in three aquarium stores in the Washington D.C. area. We found all of these specimens (*n* = 4) to be highly toxic with palytoxin or palytoxin-like compounds (range 0.5–3.5 mg crude toxin/g zoanthid). One of the most potent non-protein compounds ever discovered is present in dangerous quantities in a select species of zoanthid commonly sold in the home aquarium trade.

## Introduction

In the late 1950's, Dr. Albert H. (Hank) Banner began a program at the University of Hawaii to search for the elusive cause of ciguatera fish poisoning [1]. Dr. Philip Helfrich, a young researcher hired by Banner, began this search by investigating an entry in the Hawaiian dictionary for the “*limu-make-o-Hana*” (rough translation deadly seaweed of Hana) [2]. This legend dates back to Hawaiian antiquity with tales of Shark Gods, sacred pools, and a seaweed when applied to a warrior's spear would “bring sure death” to their enemies. The pool became “kapu” or taboo to the local Hawaiians and it was said that an ill fate would befall anyone who disturbed the sacred site. Every legend holds some basis in fact, and in 1961, Helfrich, accompanied by graduate student John Shupe, tracked down the fabled pool near the village of Mu′olea on the island of Maui and introduced to the world a new species of cnidarian zoanthid (colonial anemone) known as *Palythoa toxica* [3], [4]. This research led to the discovery of palytoxin (PLTX), one of the most potent natural products ever discovered [5]. Although much of the structural elucidation of palytoxin would be determined from less toxic, but far more abundant, species such as *Palythoa tuberculosa* [6], [7] none were ever found to be as potent as the samples collected from the tidepools at Mu′olea. Now the legendary limu appears to be exacting its ancient curse once again, but this time upon unsuspecting marine home aquarists.

**Figure 1:**
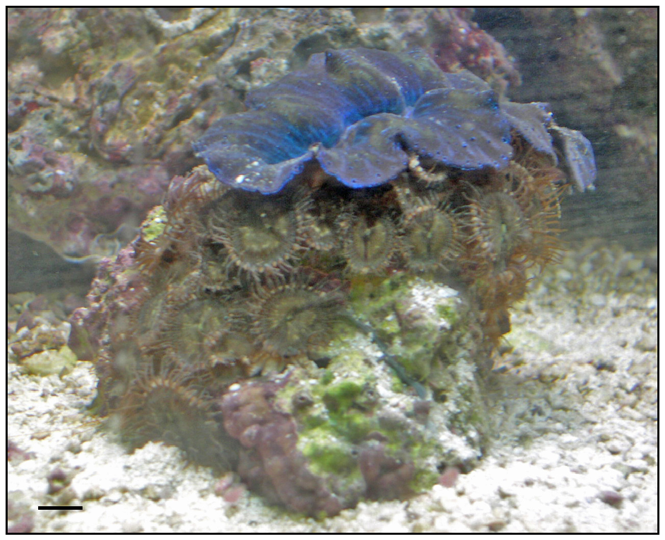
Zoanthid colony responsible for a severe respiratory reaction, collected from a home aquarium in 2008. [Referred to as VAZOA in Figure 2A and Virginia zoanthid in text]. Sample was found to contain approx. 600 µg palytoxin/g wet zoanthid. Bar represents 1 cm.

In 2007, we assisted the Georgia poison center in an investigation into a potential dermal intoxication with palytoxin from zoanthids in a home aquarium [8]. During the course of the investigation, we learned of another marine aquarium hobbyist in Virginia who had recently experienced a severe respiratory reaction while trying to eradicate brown non-descript colonies of zoanthids that had arrived as hitchhikers with live rock and were overgrowing more desirable organisms in the tank (Fig. 1) [account can be found at: http://www.reefcentral.com/forums/showthread.php?t=1083843 – accessed 12/09/10]. A sample (here called Virginia zoanthid or VAZOA) was found to contain high levels of palytoxin (600 µg crude toxin/g wet zoanthid). Zoanthids are a notoriously problematic taxonomic group [9]–[11] and it proved difficult at the time to identify to the species level the sample from the home aquarium in Virginia. Histologic evaluation of preserved polyps could only confirm that it was a *Palythoa* sp. (D. Fautin, University of Kansas, personal communication). In an attempt to determine the prevalence of palytoxin in aquarium store zoanthids, we purchased several colonies from local aquarium stores and analyzed them for palytoxin. We further performed a molecular analysis in an attempt to identify the colonies to the species level.

## Materials and Methods

### Sample preparation

Fifteen zoanthid colonies visually consistent with *Palythoa/Protopalythoa* spp. or *Zoanthus* spp. were purchased live from 3 aquarium stores in the Washington DC metro area. Samples, with associated seawater, were placed in individual glass beakers under fluorescent light for 24 hours in an attempt to induce the opening of individual polyps for photographic documentation. After photographic documentation, several polyps from each colony were removed from their supporting substrate using forceps and fine dissecting scissors and placed in 10 ml of 80% ethanol for 24 hours at 4 °C to extract PLTXs. Some of the larger polyps were split in half to facilitate extraction but no additional homogenization of tissues was performed. After extraction, polyps were removed, blotted dry, weighed, and placed in fresh 95% ethanol for DNA analysis. The total amount of tissue extracted varied depending on the size of the colony purchased and the size of the individual polyps, but the range for all samples extracted for toxin analysis in this study was 0.2–0.8 g/specimen.

### Toxin Analysis

#### Palytoxin Quantification

High performance liquid chromatography (HPLC) analysis was performed using an Agilent 1200 series HPLC system (Agilent, Wilmington, DE) with UV detection following Ciminiello et al. [12]. Samples were diluted 1∶10 using a solution of 80% acetonitrile in HPLC grade H_2_O containing 30 mM acetic acid and 30 µl was injected onto a Gemini C18 column (5 µm, 110 Å, 150 mm×2 mm) (Phenomenex, Torrance, CA). The sample was eluted using a gradient of 20% solvent A/80% solvent B to 100% solvent A over 10 min at 250 µl/min. at 30 °C [Solvent A: 95% acetonitrile in HPLC grade water, Solvent B: HPLC grade water, 30 mM acetic acid added to both]. Beginning and ending gradient conditions were maintained for 5 min. before and after the gradient run, respectively. PLTX-like compounds were detected at 263 nm and quantified through linear regression of a PLTX standard (2680 Da. from *P. tuberculosa* purchased from Wako Pure Chemical Industries, Ltd., Japan, dissolved in 50% ethanol according to manufacturer's instructions) at concentrations of 10, 5, 2.5, 1.25, 0.625, 0.3125 µg/ml. Linear regression analysis was performed using GraphPad Prism software (ver. 4.03, GraphPad Software, Inc., San Diego, CA).

#### Determination of palytoxin type

High resolution liquid chromatography mass spectrometry was used to distinguish palytoxin from 42-hydroxy-palytoxin and deoxy-palytoxin. A Thermo Exactive mass spectrometer coupled with an Accela liquid chromatography system (ThermoScientific, Waltham, MA) was used for this analysis. LC conditions were as follows: 10 ul injections were made onto a Gemini C18 column (5 µm, 110 Å, 150 mm×2 mm) (Phenomenex, Torrance, CA) and eluted with a linear gradient, as described previously, at a flow rate of 200 µl/min. Eluent was electrosprayed via the heated probe (HESI-II) into the mass spectrometer (MS). MS conditions were as follows: MS was operated in the full scan positive ion mode from 150–3000 daltons at the ultrahigh resolution setting of 100,000 @ 1 Hz. Other MS conditions are listed in Table 1. PLTX type was confirmed if the total ion chromatogram contained each of the calculated masses in Table 2 as the dominant ion in an isotopic cluster, out to two decimal places. The only predicted ion never detected for any toxin was (M+3H)^3+^. Minor PLTXs were considered present if select ions for that type were also present as the dominant ion in an isotopic cluster, typically (M+2H-H_2_O)^2+^ and (M+2H+K)^3+^, the most abundant ions present for each PLTX type in all samples.

**Table 1:** Thermo Exactive mass spectrometer conditions used for the confirmation of palytoxin, 42-hydroxy-palytoxin, and deoxy-palytoxin.

Spray Voltage (V)	3000
Capillary Temperature (°C)	250
Sheath Gas Flow Rate (arbitrary unit)	35
Aux Gas Flow Rate (arbitrary unit)	10
Sweep Gas Flow Rate (arbitrary unit)	5
Vaporizer Heater Temperature (°C)	250
Capillary Voltage (V)	30
Tube Lens Voltage (V)	130
Skimmer Voltage (V)	30
Maximum Inject Time (milliseconds)	20
Microscans	1

**Table 2:** Masses (*m/z*) used to determine palytoxin type (Top) and corresponding palytoxin detection in samples (Bottom).

Compound Name	palytoxin	42-hydroxy-palytoxin	deoxy-palytoxin
Molecular Formula	C_129_H_224_N_3_O_54_	C_129_H_225_N_3_O_55_	C_129_H_224_N_3_O_53_
**Molecular Weight**	2679.487	2696.490	2663.492
(M+H)^+^	2680.495	2697.498	2664.500
(M+2H)^2+^	1340.751	1349.253	1332.754
(M+H+K)^2+^	1359.729	1368.231	1351.732
(M+H+Na)^2+^	1351.742	1360.244	1343.745
(M+2H-H_2_0)^2+^	1331.746	1340.248	1323.749
(M+2H-2H_2_0)^2+^	1322.741	1331.242	1314.743
(M+2H-3H_2_0)^2+^	1313.736	1322.237	1305.738
(M+2H-4H_2_0)^2+^	1304.730	1313.232	1296.733
(M+3H)^3+^	894.170	899.838	888.839
(M+2H+K)^3+^	906.822	912.490	901.491
(M+2H+Na)^3+^	901.498	907.165	896.166
(M+3H-H_2_O)^3+^	888.167	893.834	882.835
(M+3H-2H_2_O)^3+^	882.163	887.831	876.832
(M+3H-3H_2_O)^3+^	876.160	881.827	870.828
(M+3H-4H_2_O)^3+^	870.156	875.824	864.824
**Sample**			
Palytoxin Standard	+++	+	-
Virginia zoanthid	+++	-	-
305.11.2	+++	-	-
306.39.2	+++	-	-
306.39.3	+++	-	-
306.37.3	+	-	+++

(Top) Calculated *m/z* values for palytoxin, 42-hydroxy-palytoxin, and deoxy-palytoxin, and their various derivatives, used to determine palytoxin type in aquarium store zoanthids using high resolution mass spectrometry.

(Bottom) Palytoxin type in highly toxic samples determined from information above. [+++] Major toxin: all ions present [except (M+3H)^3+^ - see text]. [+] Minor toxin: select ions also present as dominant ion in isotopic cluster [mainly (M+2H-H_2_0)^2+^ and (M+2H+K)^3+^]. [−] No listed ions detected as dominant ion in isotopic cluster.

### Species Identification

#### Molecular Methods

A small piece of tissue (∼10 mg) from one representative polyp from each of the 16 zoanthid samples (15 collected here plus the Virginia zoanthid sample from Deeds and Schwartz [8]) was removed using flame sterilized (with ethanol) tweezers and/or scissors and added to a sterile 1.5 ml microcentrifuge tube. DNA was extracted from tissue through the use of a DNeasy Blood and Tissue Kit (Qiagen #69506). Reagent volumes were reduced to a quarter of the volume listed in the manual (50 µl Buffer ATL with 5.56 µl of Proteinase K, followed by 55.56 µl of buffer AL and 55.6 µl of ethanol). For the wash steps, 140 µl of AW1 and AW2 were used, followed by elution with 50 µl of buffer AE. Besides this change, the manufacture's protocol was followed, with the additional step of incubating the washed filters at 37 °C for 30 min as well as the elution buffer to increase successful elution of DNA. After extraction, all samples were quantified using a Nanodrop ND 1000 Spectrophotometer (Thermo Fisher Scientific, Inc., Wilmington, DE).

Two sets of primers were used to amplify either the mitochondrial 16S (16Santla/16SBmoH) [13] or COI (LCO1490/HCO2198) [14] region. Primers were tailed with M13F-29 or M13R to simplify sequencing. Recent work has shown that zoanthid species or species group identifications can typically be achieved using a combination of these two molecular markers [11]. The PCR cocktails consisted of 6.25 µl of 10% trehalose solution (Sigma Catalog No. 90210-50 g), 2 µl of dd H_2_O, 1.25 µl of 10X PCR buffer (Invitrogen Catalog No. 10966-034), 0.625 µl of 50 mM MgCl_2_ (Invitrogen Catalog No. 10966-034), 0.125 µl of 10 µM of each of the F/R primers, 0.062 µl of 10 mM dNTPs (New England Biolabs Catalog No. N0447L), and 0.060 µl of Platinum Taq (5 U/µl, Invitrogen Catalog No. 10966-034) and 1 µl of undiluted DNA template per reaction, according to Ivanova et al. [15]. An Eppendorf Mastercycler® ep gradient S Thermocycler was used for the PCRs with the following conditions: 95°C for 1 min, 35 cycles of 95°C for 1 min, 50°C for 40 sec (for 16S, 40°C for COI), and 72°C for 1.5 min, with a final extension at 72°C for 7 min.

All products were verified using pre-cast 1% E-gel 48 agarose gels (Invitrogen Catalog No. G8008-01) according to manufacturer's protocols with the E-Base® Integrated power supply (Invitrogen Catalog No. EB-M03). Gels were run for 15 min and then visualized using a Bio-Rad gel documentation system (Gel Doc 2000, Hercules, CA). The gel was also photographed with this instrument and the picture retained for records.

Successfully amplified products were purified with Exosap-IT (USB, Cleveland, OH) by adding 2 µl of Exosap-IT to 5 µl of PCR product, incubating at 37 °C for 15 minutes followed by 15 minutes at 80 °C.

At least two sequencing reactions (in the forward and reverse direction) were run on each sample at the Smithsonian Institution's Laboratories of Analytical Biology (Suitland, MD, USA). Sequencing reactions consisted of 0.5 µl of BigDye® Terminator v3.1 (Applied Biosystems), 1.75 µl of 5X sequencing buffer (Applied Biosystems), 0.5 µl of primer (either M13F-29 or M13R), 6.25 µl of molecular grade water for a total of 9 µl to which 1 µl of cleaned up PCR product was added. Sequencing reactions were conducted on an Eppendorf Mastercycler® ep gradient S Thermocycler with the following conditions with the following conditions: 95°C for 2 min, 30 cycles of 95°C for 30 sec, 50°C for 15 sec, and 60°C for 4 min, followed by a 10°C hold. Reactions were cleaned using a sephedex column, and then dried down. Finally 10 µl of Hi-Di formamide (Applied Biosystems #4311320) was added to each sample and heated at 95°C for 2 min. Samples were then sequenced on an ABI 3730xL sequencer (Applied Biosystems, Foster City, CA), which was maintained according to manufacturer's specifications. Resulting sequences were edited in Sequencher 4.9 (Gene Codes Corp., Ann Arbor, MI).

#### Phylogenetic Analysis

Nucleotide sequences of 16S and COI from samples were manually aligned with previously published 16S and COI sequences from various zoanthid species representing the genera *Palythoa*, *Zoanthus* and *Isaurus*. These published sequences were the top matches to the unknown samples using the BLAST algorithm [16] to search the National Center for Biotechnology Information (NCBI) database located at http://www.ncbi.nlm.nih.gov/. Out-group sequences for both 16S and COI trees were from the genera *Hydrozoanthus* (within suborder Macrocnemina).

All alignments were inspected by eye and manually edited. All ambiguous sites of the alignments were removed from the dataset for phylogenetic analyses. Consequently, two alignment datasets were generated: 1. 817 sites of 30 sequences (16S) and 2. 461 sites of 26 sequences (COI). The alignment data are available on request from the corresponding author, and also at the webpage http://web.me.com/miseryukyu.

For the phylogenetic analyses of the two alignments, the same methods were applied independently. Alignments were subjected to analyses with maximum-likelihood method (ML) with PhyML [17] and neighbor-joining (NJ) method with CLC Free Workbench 3 [18]. PhyML was performed using an input tree generated by BIONJ with the general time-reversible (GTR) model [19] of nucleotide substitution incorporating invariable sites and a discrete gamma distribution (eight categories) (GTR + I + Γ). The proportion of invariable sites, a discrete gamma distribution, and base frequencies of the model were estimated from the dataset. PhyML bootstrap trees (1000 replicates) were constructed using the same parameters as the individual ML tree. The distances were calculated using a Kimura's 2-parameter model [20]. Support for NJ branches was tested by bootstrap analysis [21] of 1000 replicates.

Bayesian trees were reconstructed by using the program MrBayes 3.1.2 under the GTR model [22]. One cold and three heated Markov chain Monte Carlo (MCMC) chains with default-chain temperatures were run for 20 million generations, sampling log-likelihoods (InLs), and trees at 100-generation intervals (20,000 InLs and trees were saved during MCMC). The first 15% of all runs were discarded as “burn-in” for all datasets. The likelihood plots for both datasets also showed that MCMC reached the stationary phase by this time (PSRF = 1.000 for both data sets). Thus, the remaining 170,000 trees (17 million generations) of 16S and COI were used to obtain posterior probabilities and branch-length estimates, respectively.

## Results and Discussion

### Toxin Analysis

**Figure 2:**
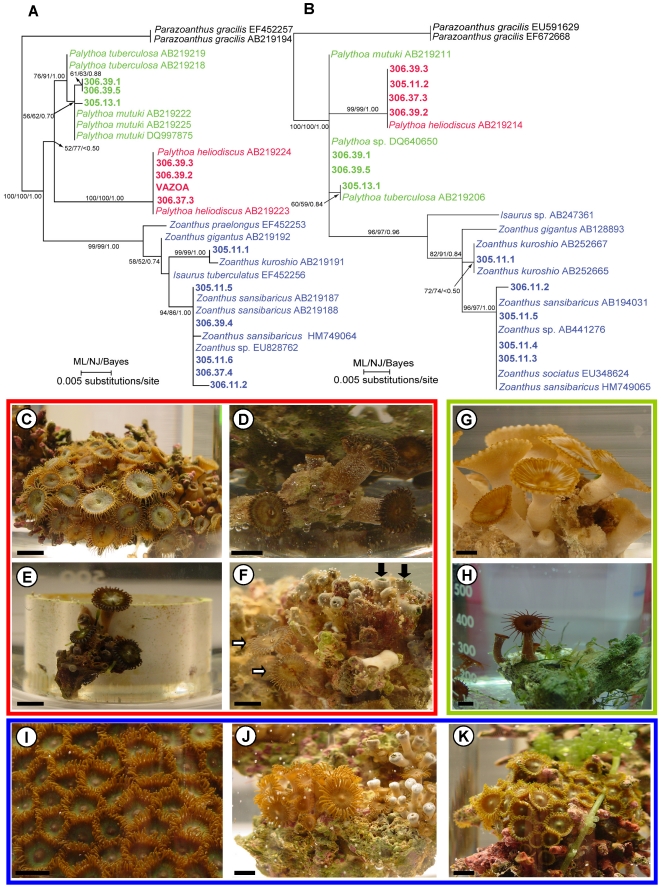
Species identification of zoanthid samples collected from aquarium stores. Maximum-likelihood (ML) phylogenies of zoanthids showing both the 16S [A] and COI [B] data sets. Support above branches: ML bootstraps/Neighbor-joining bootstraps/Bayesian posterior probabilities. [C-K] Representative specimens for *Palythoa* spp. (both toxic and non-toxic/weakly-toxic) and *Zoanthus* spp. (all non- or weakly-toxic) collected from local aquarium stores including: [C] sample 305.11.2, [D] sample 306.37.3, [E] sample 306.39.2, [F] samples 306.39.3 [open arrows] and sample 306.39.4 [closed arrows], [G] sample 305.13.1, [H] sample 306.39.1, [I] sample 305.11.6, [J] sample 305.11.5, and [K] sample 305.11.3. [Red Box] Visually and genetically consistent with *Palythoa* spp. and containing high concentrations of palytoxins (500–3500 µg/g wet zoanthid). [Green Box] Visually and genetically consistent with *Palythoa* spp. but non- or weakly-toxic, and [Blue Box] visually and genetically consistent with *Zoanthus* spp., and non- or weakly-toxic. For [C-K], bar represents 1 cm.

After our initial finding of a highly toxic *Palythoa* sp. in a home aquarium in Virginia [sample “Virginia zoanthid”, described in Deeds and Schwartz [8], (Fig. 1)], we visited a local aquarium store known to carry zoanthids (aquarium store #1) and purchased every visually distinct variety of zoanthid they had for sale (*n* = 7). These included specimens visually consistent with both *Palythoa/Protopalythoa* spp. and *Zoanthus* spp. (according to [23]). Of these, only one proved to be as toxic as the sample from the Virginia home aquarium (sample 305.11.2) (Table 3). Among the specimens purchased from aquarium store #1, only sample 305.11.2 was morphologically similar to the Virginia zoanthid sample (Fig. 1, Fig. 2C). With this information, two additional aquarium stores were visited (aquarium store #2 and #3), targeting specimens that were visually consistent with our two toxic *Palythoa* sp. samples. These stores were identified as the most likely in the area to carry zoanthids based on discussions with local marine aquarium hobbyists. Between the two stores, eight additional specimens were acquired. Of these, three were found to be highly toxic (Table 3). The first sample consisted of a few individual polyps growing on a small fragment of coral at the bottom of a large zoanthid display tank (sample 306.37.3) (Fig. 2D) in aquarium store #2. The small specimen was located at the bottom of the tank, under a display rack, but was retrieved by the store owner and sold for a minimal price. The last two samples, acquired from aquarium store #3, were either growing as disperse polyps among a colony of another zoanthid species (sample 306.39.2), or as a few random polyps growing on the side of a small piece of plastic pipe sitting on the bottom of another display tank (sample 306.39.3) (Fig. 2E,F respectively).

**Table 3:** Sample information for specimens analyzed to determine prevalence of palytoxins in commercially available zoanthids.

Sample ID	16S	COI	Store #	Toxin	Concentration	Identification*
VAZOA	X		-	PLTX	613 µg/g	*Palythoa heliodiscus*
305.11.2		X	1	PLTX	515 µg/g	*Palythoa heliodiscus*
306.37.3	X	X	2	deoxy-PLTX	3515 µg/g	*Palythoa heliodiscus*
306.39.2	X	X	3	PLTX	1164 µg/g	*Palythoa heliodiscus*
306.39.3	X	X	3	PLTX	1037 µ µg/g	*Palythoa heliodiscus*
305.13.1	X	X	1	-	Weakly Toxic	*Palythoa mutuki*
306.39.1	X	X	3	-	Non-Toxic	*Palythoa mutuki*
306.39.5	X	X	3	-	Non-Toxic	*Palythoa mutuki*
305.11.3	X	X	1	-	Weakly Toxic	*Zoanthus sansibaricus* **
305.11.4		X	1	-	Non-Toxic	*Zoanthus sansibaricus* **
305.11.5	X	X	1	-	Weakly Toxic	*Zoanthus sansibaricus* **
305.11.6	X		1	-	Non-Toxic	*Zoanthus sansibaricus* **
305.11.1	X	X	1	-	Non-Toxic	*Zoanthus kuroshio*
306.11.2	X	X	2	-	Non-Toxic	*Zoanthus sansibaricus* **
306.37.4	X		2	-	Non-Toxic	*Zoanthus sansibaricus* **
306.39.4	X		3	-	Non-Toxic	*Zoanthus sansibaricus* **

*closest match from publically available sequences (Fig. 2).

**also matched single COI sequence for *Zoanthus sociatus* (Fig. 2).

Weakly-Toxic - defined as possessing a hemolytic component that was inhibited with an anti-PLTX antibody but too low in concentration to confirm as a PLTX by additional chemical means (methods described in [8]).

Non-Toxic – defined as no detectable toxic activity using a hemolysis neutralization assay and no HPLC peak at 263 nm consistent with a PLTX standard.

**Figure 3:**
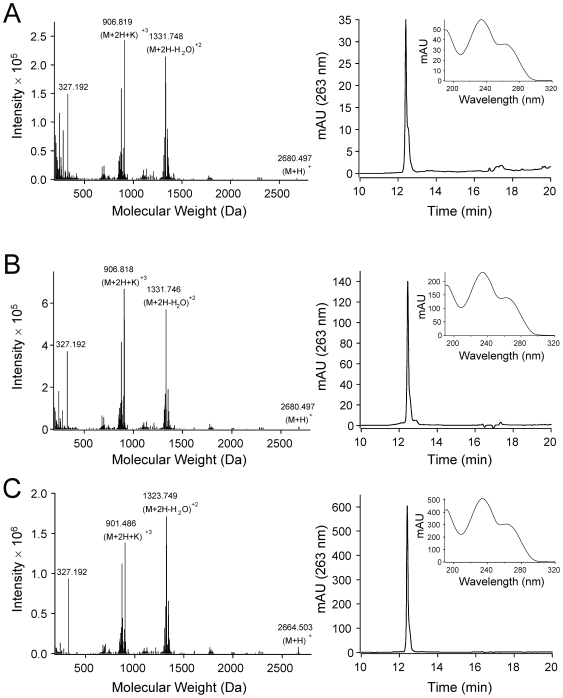
Toxin analysis of zoanthid samples collected from aquarium stores. High resolution mass spectrometric (left column) and high performance liquid chromatographic (right column) analysis of [A] palytoxin standard, from *P. tuberculosa*, purchased from Wako Pure Chemicals, Tokyo, Japan, [B] sample 305.11.2 (Figure 2C), purchased from aquarium store #1, which contained approx. 500 µg palytoxin/g wet zoanthid and [C] sample 306.37.3 (Figure 2D), purchased from aquarium store #2, which contained approx. 3500 µg deoxy-palytoxin/g wet zoanthid. For all 3, the inset graph in the right column is the UV spectra for the HPLC peak at 12.4 minutes. All samples contained a 327 Da. fragment and UV maxima at 233 and 263 nm, which are characteristic of palytoxins.

Using high performance liquid chromatography (HPLC) with UV detection, with quantification against a PLTX standard, we found that all four of the purchased zoanthids that were visually consistent with the Virginia home aquarium specimen were themselves highly toxic (approx. 500-3500 µg crude toxin/g wet zoanthid) (Fig. 3, Table 3). In our previous study, we found the Virginia home aquarium sample to contain approx. 600 µg crude toxin/g wet zoanthid [8]. For comparison, the original Hawaiian *P. toxica* collections in the 1960's yielded approximately 300 µg pure toxin/g wet zoanthid [2]. Using electrospray ionization liquid chromatography mass spectrometry (ESI/LC/MS) with a high resolution mass spectrometer, for confirmation of PLTX type, four of the samples were shown to contain only palytoxin (Virginia zoanthid, 305.11.2, 306.39.2, 306.39.3) while the last (306.37.3) contained primarily deoxy-palytoxin with a lesser amount of palytoxin (Table 2). Minor PLTXs could not be quantified due to a lack of chromatographic resolution. None were found to contain 42-hydroxy-palytoxin, the primary toxin recently reported from samples collected in the 1990s from the original Hana tidepools from, presumably, *P. toxica* [24]. As expected, our PLTX standard, isolated from *P. tuberculosa*, contained primarily palytoxin with a minor amount of 42-hydroxy-palytoxin [22] (Table 2). Several deoxy-palytoxins have been previously described: one as a minor toxin from *P. tuberculosa* [25], and more recently, several from cultures of the dinoflagellate *Ostreopsis* cf. *ovata* and *Ostreopsis ovata* (Rossi et al. [26], therein called ovatoxin-b, and Ciminiello et al. [27], therein called ovatoxins d and e, respectively). This is the first report of a deoxy-palytoxin being the primary toxin from any zoanthid. 42-hydroxy-palytoxin has been shown to be similar in potency with palytoxin [24]. None of the described deoxy-palytoxins have been assessed for potency. In this study, the position of deoxygenation was not determined, therefore the exact relationship of the deoxy-palytoxin described here with those previously described could not be established. All of the other samples collected (*n* = 11), both additional *Palythoa* spp. and other specimens visually and genetically (see below) consistent with *Zoanthus* spp., were either non-toxic or weakly-toxic (i.e. shown to have an extractable hemolytic component with properties consistent with PLTX but too low in concentration to confirm as PLTX by other chemical means – detailed methods can be found in [8]).

### Species Identification

Mitochondrial 16S and COI markers were successfully generated for 10 of the 16 specimens from this study (Table 3). For the remaining six specimens either 16S or COI sequences were generated. All failed sequences were attempted at least twice. Phylogenetic trees generated using both markers showed the same result that all of our toxic samples (*n* = 5) formed a closely related clade that was distinct from the additional non- weakly-toxic *Palythoa* spp. (*n* = 3) and the non- weakly-toxic *Zoanthus* spp. (*n* = 8) (Fig. 2A,B). The most closely related species to our toxic specimens based on available molecular data is *Palythoa heliodiscus*. It should be noted that relatively few of the described species of zoanthids have been analyzed genetically. To our knowledge, no molecular data is available for *P. toxica*. We were able to acquire a sample of the *P. toxica* type specimen from the Bishop museum in Honolulu, Hawaii, but due to its age and original fixation method were unable to extract any DNA. Our request to the local governing bodies who now hold stewardship over the type location at Mu′olea to collect a new sample for genetic analysis were denied, so we could not determine the relationship between our samples and *P. toxica*. However, pictures of *P. toxica* [3] are consistent with both our specimens as well as available pictures of *P. heliodiscus* [28]. To our knowledge, *P. heliodiscus* has never been tested for toxicity.

### Conclusions

Some of the deadliest toxins known to man come from the sea. Maitotoxin produced by tropical single celled dinoflagellates of the genus *Gambierdiscus* has an intraperitoneal (IP) lethality (in mice) of 50 ng/kg [29]. Meaning 1 gram of toxin can kill 1 billion mice. It is the most potent non-peptide natural product ever discovered. Palytoxin comes in second with an IP mouse lethality of 300 ng/kg [30]. Even though our typical sample size for zoanthids collected in this study was small (0.2–0.8 g/sample), we calculate that we extracted enough crude toxin from these combined samples to kill 300,000 mice (approx. 2 mg crude toxin calculated by HPLC, standard mouse size of 20 g).

Palytoxins, along with several structurally related compounds (e.g. ostreocins [31] and ovatoxins [26]–[27], [32] produced by select marine dinoflagellates) have garnered significant recent attention due to the fact that they are one of the few marine toxins that pose a risk to humans through ingestion (consumption of contaminated seafood), inhalation (exposure to palytoxin containing aerosols), and dermal (exposure to select marine zoanthids) routes of exposure [33].

Numerous poisonous and venomous animals are available at your local pet store. Animals imported for the pet/aquarium trade are regulated based on their CITES (Convention on International Trade in Endangered Species of Wild Fauna and Flora) status and not based on potential toxicity. Venomous lionfish (family Scorpaenidae) [34], and alkaloid containing poison dart frogs (family Dentrobatidae) [35] are commonplace. Although more rare, highly venomous (with tetrodotoxin) blue ringed octopuses (*Hapalochlaena* spp.) [34] can occasionally be found in home aquaria. Furthermore, importation of whole tetrodotoxin containing puffer fish (family Tetraodontidae), which are prohibited for importation (whole) as food [36], have no such restrictions if they are imported as pets.

There is great debate among marine aquarium enthusiasts on the hazards of keeping zoanthids. Some literature warns that all zoanthids are toxic [23], while many hobbyists claim that they have been handling these organisms for years without incident (on-line accounts can be found at sites such as www.reefcentral.com). Well documented cases of human poisonings due to exposure to zoanthids in home aquaria are limited, but detailed exposure accounts with confirmation of toxin presence do exist for inhalational [8] and dermal [37] exposures. Additional case reports based on symptoms alone can also be found in the literature [8], [38]–[39]. This is the first report to our knowledge that documents both high PLTX presence and phylogenetic identity for commercially available zoanthids from aquarium stores.

It is often difficult to determine the species, let alone geographic origin, for many of the organisms sold in the home aquarium trade. In the case of zoanthids, most specimens are sold under common names such as “button polyps”, “sun polyps”, or “yellow/orange or green zoas”; furthermore, many enthusiasts and websites selling zoanthids use colloquial names such as “watermelon”, “scorpion”, “people eaters”, “sunset” palys or zoas, etc. Discussions with the owner of aquarium store #1 revealed that he typically acquired zoanthids though the purchase of mixed containers of “frags” (i.e. coral or rock fragments containing zoanthid colonies) from either importers or wholesalers who typically reported them to be wild caught and of Indo-Pacific origin but with no other accompanying documentation confirming the geographic origin or identity of the specimens. Making the issue of tracing the origin of commercial zoanthids more difficult is the fact that many marine aquarium enthusiasts commonly fragment their colonies and further exchange them outside of retail sale. All of this uncertainty makes it difficult to provide a concise message to aquarium hobbyists on the hazards of keeping this group of organisms. During this investigation, we found that many of the zoanthids commonly sold in the home aquarium trade are non-toxic or weakly-toxic, but a highly toxic variety of *Palythoa* (possibly *P. heliodiscus* or *P. toxica*) is indeed available. It often occurs as a tank contaminant and can be unintentionally introduced with more desirable species or on live rock. Unfortunately, due to the unavailability of molecular data for *P. toxica* (the *limu make o Hana* from Hawaiian folklore), we could not determine its relationship to either the toxic specimens from our study or to *P. heliodiscus*, which available molecular data suggests our toxic specimens are. Hopefully, one day the type location on the island of Maui will be made available for sampling again and the true identity of *P. toxica* can be determined. Regardless, we have shown that a *Palythoa* sp. just as toxic as the legendary *limu* can not only be found outside of the tidepools at Mu′olea, it is available in retail commerce and present in home aquaria, with the owners often unaware of the deadly poisons they are being exposed to.
